# Regional specification and complementation with non-neuroectodermal cells in human brain organoids

**DOI:** 10.1007/s00109-021-02051-9

**Published:** 2021-03-02

**Authors:** Yoshiaki Tanaka, In-Hyun Park

**Affiliations:** 1 Department of Genetics, Yale Stem Cell Center, Child Study Center, Yale School of Medicine, New Haven, CT 06520 USA; 2 Present Address: Department of Medicine, Maisonneuve-Rosemont Hospital Research Center, University of Montreal, Montreal, QC H1T 2M4 Canada

**Keywords:** Non-neuroectodermal cells, Organoids, Organoid technologies, Brain organoids, Cortical organoids, hESCs, Neurodevelopment, Endothelial cells, Microglia, Neurons

## Abstract

Along with emergence of the organoids, their application in biomedical research has been currently one of the most fascinating themes. For the past few years, scientists have made significant contributions to deriving organoids representing the whole brain and specific brain regions. Coupled with somatic cell reprogramming and CRISPR/Cas9 editing, the organoid technologies were applied for disease modeling and drug screening. The methods to develop organoids further improved for rapid and efficient generation of cerebral organoids. Additionally, refining the methods to develop the regionally specified brain organoids enabled the investigation of development and interaction of the specific brain regions. Recent studies started resolving the issue in the lack of non-neuroectodermal cells in brain organoids, including vascular endothelial cells and microglia, which play fundamental roles in neurodevelopment and are involved in the pathophysiology of acute and chronic neural disorders. In this review, we highlight recent advances of neuronal organoid technologies, focusing on the region-specific brain organoids and complementation with endothelial cells and microglia, and discuss their potential applications to neuronal diseases.

## Introduction

The central nervous system (CNS) comprises the brain and spinal cord, and mediates most activities in body and mind processes. Although surgically isolated and postmortem brains from the patients are invaluable resources to study the pathology of the neuronal diseases, access to these human brain samples is very limited due to ethical and practical reasons. Thus, mouse models have been widely applied to molecular studies in the brain development and the drug response. However, in genetic and molecular levels, rodents differ from humans, displaying a vastly dissimilar developmental program. Brain organoids (also called as cerebral organoids) are three-dimensional (3D) brain models in a laboratory dish. They have arisen as innovative model systems to study human brain development and diseases. In this review, we will provide overview how the brain organoids are produced, especially focusing on the region-specific brain organoids. We will also discuss the challenges in brain organoid fields and recent implementation of non-neuronal cells in brain organoids to complete organization of brain composition.

## Development of brain organoids

When human pluripotent stem cells (hPSCs) are cultured in the 3D aggregates, they differentiate into various brain cell types and spontaneously organize into structures recapitulating the developing human brain. hPSC-derived brain organoids represent the promising resources to investigate molecular mechanisms of brain development and disorders. At gastrulation stage, the primary central nervous system (CNS) appears as a neural plate that is composed of embryonic neuroectoderm cells [1]. The neural plate converges toward the dorsal midline of the embryo to form a neural groove that is subsequently enclosed to build a tubular structure. In the third and fourth weeks of gestation, the developing neural tube is divided into three major vesicles: forebrain (prosencephalon), midbrain (mesencephalon), and hindbrain (rhombencephalon). The forebrain further develops into two subdivisions: telencephalon and diencephalon. These primary brain vesicles form segmental anteroposterior structures that constitute prosomeres 1-3 (p1-3) of diencephalon, mesomeres 1 and 2 (m1 and 2) of midbrain, and isthmus (r0) and rhombomeres 1-11 (r1-11) of hindbrain [2]. The neuromeric compartments eventually give rise to the individual brain regions, such as the cortex, thalamus, and cerebellum. The most posterior portion of the neural tube serves as the central canal of the spinal cord.

**Fig. 1:**
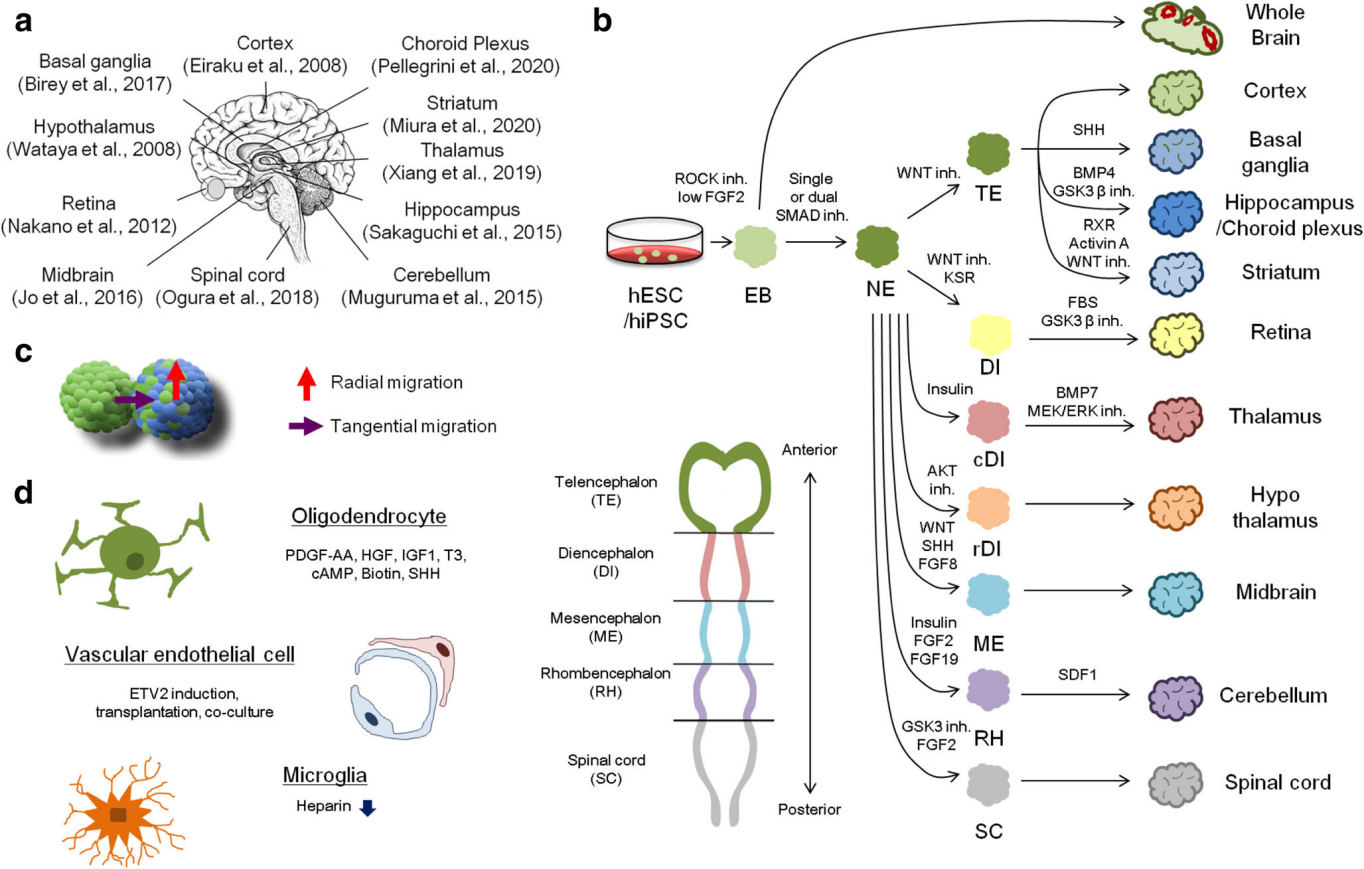
Region-specific 3D brain culture systems from human pluripotent stem cell. **a** Recent accomplishment of region-specific brain organoids. **b** Schematic procedures of whole and region-specific brain organoid derivations. **c** Fused assembloids as models for the study of radial and tangential neuronal migration. **d** Compensation of absent cell types in conventional brain organoid protocols

Since the serum-free floating culture of embryonic body (SFEB) has provided a blue-print to form 3D structures of brain organoids from Dr. Sasai’s group [3], researchers have been inspired to address how brain tissue is differentiated and structured, and to optimize the culture systems for hPSC-derived organoids to more faithfully recapitulate in vivo brain. Eiraku further improved the SFEB method to expedite the EB formation (SFEBq) that was applied to generate 3D structures that recapitulate the early steps of human corticogenesis [4]. The formation of embryonic bodies can be archived under low-adhesion culture condition, and is an initial step to form the brain organoid. EB harbors a remarkable developmental capacity to form neuronal tube–like structures in the presence of a low amount of FGF2 and substantially differentiate into neurons and glia cells by withdrawal of FGF2. Using this intrinsic property of EB, Lancaster and colleagues [5] pioneered the generation of whole brain organoids that display discrete regional identities ranging from the forebrain to retina with a supportive extracellular matrix Matrigel. Importantly, Kasoshima and colleagues defined the self-organization of the cortical organoids with the axial identity and region-specific morphogenesis [6]. The whole brain organoids enable us to address divergence and inter-dependency of various brain regions and can be applicable to cephalic disorders affecting a large portion of the brain, such as anencephaly and microcephaly. However, the developmental process of whole brain organoids is stochastic, resulting in high heterogeneity across the individual organoids and among the hPSC lines [7–9]. Unlike the un-patterned methodology, the combinatorial application of signaling modulators and growth factors enhances to guide the human organoids into specific areas of CNS: cortex [4], basal ganglia [10], hippocampus [11], choroid plexus [12], striatum [13], thalamus [14], retina [15], hypothalamus [16], midbrain [17], cerebellum [18], and spinal cord [19] (Table 1 and Fig. 1a). In the following sections, we highlight the recent advances of the region-specific brain organoids and the protocol variability, and discuss the potential applications for neuronal disease modelings.

**Table 1:** List of signaling modulators and potential actions for regional specification

Signaling modulation	Representative morphogens/molecules	Potential actions
ROCK inhibition	Y27632	EB formation and differentiation, cell viability
Low FGF2 stimulation	FGF2	EB formation and differentiation, cell viability
TGFf inhibition	SB-431542	Neuroectodermal patterning
BMP inhibition	LDN-193189, dorsomorphin	Neuroectodermal patterning
WNT inhibition	IWR-1-endo, XAV939, IWP2	Neuroectodermal patterning, dorsal patterning
SHH inhibition	Cyclopamine A	Dorsal patterning
SHH stimulation	recombinant SHH, purmorphamine, SAG	Ventral patterning
Retinoid X receptor stimulation	SR11237	LGE patterning
Activin receptor stimulation	Activin A	Lateral patterning
GSK3 inhibition	CHIR99021	Dorsomedial patterning, hem induction
BMP4 stimulation	BMP4	Dorsomedial patterning, choroid plexus induction
MEK-ERK inhibition	PD0325901	Caudal forebrain patterning by inhibiting midbrain patterning
BMP7 stimulation	BMP7	Caudal forebrain patterning
Insulin stimulation	Insulin	Caudal forebrain patterning
AKT inhibition	Inhibitor VIII	Rostral forebrain patterning
FGF8 stimulation	FGF8	Organization of mesencephalic and rhombencephalon boundary
FGF2 stimulation	FGF2	Caudal patterning
FGF19 stimulation	FGF19	Dorsoventral axis patterning
RA stimulation	Retinoic acid	Dorsoventral axis patterning

## Region-specific brain organoids

### Dorsal and ventral cortex

EBs are forced to develop into the early neuroectoderm (NE) with single or dual SMAD inhibition (e.g., SB-431542 or LDN-193189) that blocks mesenchymal differentiation (Fig. 1b). The neuroepithelial rosette spontaneously arises and can be guided toward specific brain divisions with respect to dorsoventral and anterior-posterior axis. Telencephalon (TE) development is facilitated by canonical WNT signaling inhibition (e.g., XAV939 or IWP2) and spontaneously gives rise to the dorsal cortex [4, 8, 20]. To date, various types of protocols for the cortical organoids have been proposed or modified for their efficient and rapid generation by different combinations of inhibitors for WNT, TGFβ, BMP, and sonic hedgehog (SHH) pathways (highlighted in [21]). These public protocols show varied levels of directed differentiation to the cortex with the combination of the small molecules [22]. Recently, we demonstrated by systematic comparison of single-cell transcriptome data that these diverse protocols produced the brain organoids that exhibit similar cell repertoires, but unique preference of developmental trajectories at early stage [21]. An independent integrative analysis by Bhaduri and colleagues also showed that the cell type composition is similar in spite of the difference of protocols and starting stem cell lines [22]. The specification of basal forebrain regions is achieved by stimulating TE aggregates with recombinant SHH or its agonists (e.g., purmorphamine or smoothened agonist (SAG)) [10, 20, 23]. The basal forebrain organoids were also generated by various groups and termed as subpallium spheroid [10], medial ganglionic eminence (MGE) organoid [20], or ventral cerebral organoid [23]. Inhibitory GABAergic interneuron is a major constitution of the basal ganglia. The interneuron migration and integration into the cortical neuronal circuits are essential events for the establishment of primary excitatory and inhibitory balance in the healthy brain. The fusion of the basal organoids and the cortical organoids recapitulated the tangential interneuron migration from ventral and dorsal forebrain in vitro [10, 20, 23] (Fig. 1c). The migrated interneurons are mainly localized outside of the ventricular zone–like area in the cortical organoids and exhibit synaptic connections with cortical neurons to construct the excitatory-inhibitory neural networks that maintain proper emotional and physical activities. However, the balance between excitatory and inhibitory signals is often disrupted in autism spectrum disorders and schizophrenia. The loss of inhibitory or excessive excitatory signal leads to defects of learning and cognition in these patients. The dorsal and ventral forebrain organoids from patient-derived hPSCs or mutant hESCs allow us to address not only abnormalities in neurogenesis and activity in both excitatory and inhibitory neurons, but also defects of migration and interaction with the fusion experiment. Therefore, the dorsal and ventral forebrain organoids are pertinent for the modeling of schizophrenia and autism spectrum disorders.

### Striatum organoid

The striatum integrates neuronal signals from multiple cortical regions and connects downstream neuronal circuits in the basal ganglia to control voluntary movement, emotion, and cognitive functions. The striatum mainly originated from the ventral telencephalon, in particular the lateral ganglionic eminence (LGE). Brain organoids specified to the striatum area were recently derived by prolonged culture of LGE-like cell aggregates that are generated by retinoid X receptor stimulation (e.g., SR11237), Activin A stimulation, and WNT inhibition [13]. The LGE-like organoids preferentially express a unique marker, GSX2, while a MGE marker, NKX2-1, was rarely expressed in the LGE organoid. The striatum organoids produced striatal medium spiny neurons with plentiful dendritic spines, slow-ramp depolarization, and resting membrane potential. Fusion with the cortical organoid demonstrated that unidirectional glutamatergic neuronal projections are caused from the cortex side and form synaptic connections with GABAergic medium spiny neurons in the striatum organoid. Phelan-McDermid syndrome (PMS) is one of the neurodevelopmental disorders with severe impairment of learning and speech, and caused by a genomic deletion on chromosome 22q13.3. PMS patients display a smaller volume of the striatum and aberrant synaptic transmission in striatal neurons. Although the patient-derived striatum organoids did not show a different size from the healthy controls, the cortico-striatal assembloids exhibited higher calcium signals in the striatum side. The striatum organoid may give molecular insights not only into brain development but also into the pathogenesis of neurological diseases.

### Hippocampus and choroid plexus organoid

The hippocampus is a part of the limbic system and plays a main role in learning, memory, and emotion and is vulnerable to metabolic and cytotoxic insults including traumatic injury, ischemia, and aging. Atrophy and hyperexcitability of hippocampus areas are frequently detectable in patients diagnosed with Alzheimer’s disease (AD) or schizophrenia, and are associated with memory loss and cognitive impairment. Whereas the cortex and basal ganglia are derived from dorsal (dorsal pallium) and ventral TE (subpallium), respectively, hippocampus arises from dorsomedial TE (medial pallium). The medial patterning of TE aggregates can be induced by transient exposure (3 days) of the GSK3β inhibitor and BMP4 to modulate the dorsal midline formation [11]. Medial pallium is anatomically adjacent to two dorsalization organizers: choroid plexus and cortical hem. Treatment of the GSK3β inhibitor activates Wnt signaling to induce choroid plexus–like tissue, whereas cortical hem–like tissue is derived by BMP4 stimulation. The hippocampus organoids successfully exhibit continuous structures of choroid plexus, cortical hem, and medial pallium tissues. The current hippocampus organoid successfully produces dentate gyrus (DG) granule and CA3 pyramidal–like neurons and recapitulates the early developmental stage of the embryonic hippocampus, but fails to generate CA1 pyramidal–like neurons. To achieve the maturation and long-term culture, further improvement and investigation of the organoid culture system are needed.

An alternative protocol was recently established to direct the organoids more toward the choroid plexus region with higher concentrations of BMP4 [12]. The choroid plexus organoids form tight barriers that prevent the entry of dopamine but selectively transport its precursor, levodopa, from the medium into the internal fluid. The colorless fluid inside the organoids resembles protein components with cerebrospinal fluid (CSF) in vivo. Overall, the choroid plexus organoids are promising models to test drug permeability and to investigate CSF production during embryonic and postnatal brain development.

### Thalamic and hypothalamic organoid

The thalamus and hypothalamus are located under the cerebral cortex and develop from the caudal and rostral diencephalon region, respectively [14, 16]. In particular, the nuclei of the dorsal thalamus are derived from the embryonic prosomere 2 segment [2]. Exogenous insulin promotes the caudal diencephalon (cDI) development. Following MEK-ERK signaling inhibition (e.g., PD0325901) to cDI aggregates helps the prevention of an excessive caudalization toward mesencephalon. BMP7 stimulation accelerates the commitment of the cDI aggregates into the thalamic cell fate. The thalamus relays motor and sensory information to the cortex by reciprocal neuronal projections. Thalamic atrophy or structural abnormality is observed in motor impairment diseases, such as idiopathic Parkinson’s disease (PD), frontotemporal dementia (FTD), and amyotrophic lateral sclerosis (ALS) [24, 25]. For example, cortico-basal ganglia-thalamo-cortical (CBGTC) or cortico-striatal-thalamo-cortical (CSTC) neuronal circuit is essential for voluntary motor movement, and impaired in PD patient’s brain [26]. The excitation of thalamo-cortical projection neurons ignites movement execution and is dampened by the output from the basal ganglia. The loss of dopamine signal in PD causes the miscommunication between the BG and the thalamus [27]. The assembly of the thalamic organoid with the cortical organoid successfully created extensive cortico-thalamic and thalamo-cortical axon projection and may be potentially available to understand the pathology of these neuronal diseases [14]. The coculture with rat cortical slice shows the radial extension of thalamic neurites from the organoid into the specific cortical layer and eventually marginal zone [28]. In addition, the availability of basal ganglia and striatum organoids potentially offers in vitro modeling of CBGTC and CSTC loops, and application for pathophysiology study and drug testing of PD.

NE aggregates robustly differentiate into the rostral diencephalon (rDI) under growth factor–free suspension culture. Because of the inhibitory effect of insulin signaling on the rostralization, the inhibitors for AKT signaling (e.g., inhibitor VIII), which is an insulin-downstream pathway, promote hypothalamic differentiation [16, 29]. An alternative approach employs the activation of SHH and canonical WNT signaling for hypothalamic neurogenesis, respectively [30]. The hypothalamus is responsible for the homeostasis by governing physiological and behavioral processes with the endocrine and autonomic nervous system. The hypothalamic organoid produces RAX-expressing hypothalamic progenitor cells that substantially give rise to broad types of hypothalamic neuropeptidergic and hormone-releasing neurons including arginine vasopressin, corticotropin-releasing hormone (CRH), and thyrotropin-releasing hormone [16, 29]. Furthermore, the SHH stimulation to the hypothalamic organoid enhances the regionalization into the rostral-ventral hypothalamus, where RAX-expressing neurons differentiate into TH^+^/NKX2.1^+^ dopaminergic neuron and agouti-related protein–releasing neurons [16].

The pituitary gland is another homeostatic center in the brain and anatomically lies beneath the hypothalamus. In particular, the anterior pituitary regulates growth, stress, and sexual reproduction by synthesizing and releasing their related hormones and is governed by regulatory hormones from the hypothalamus via the hypophyseal portal system. The aberrant hypothalamic-pituitary axis gives rise to the disorders of metabolism and fertility, such as obesity, hypothyroidism, and hypogonadism. Despite the functional and spatial relationship, the anterior pituitary emerges from a different origin from the hypothalamus. The pituitary organogenesis is initiated by the formation of Rathke’s pouch that arises from an upward invagination of non-neuronal oral ectoderm adjacent to the hypothalamic origins in the developing embryo. Early BMP4 exposure to the ventral hypothalamic organoids facilitates the superficial formation of the oral ectoderm layer that subsequently invaginates to form Rathke’s pouch [31]. The pituitary organoid exhibits corticotrophs and somatotrophs with the secretion ability of adrenocorticotropic hormone (ACTH) and growth hormone (GH) by the exogenous stimulation of CRH and growth hormone–releasing hormone (GHRH), respectively. Overall, the hypothalamic and pituitary organoids are promising platforms to investigate the development and defects of the hormone cascade.

### Retinal organoid

The retina is the extension of the CNS and originated from the optic vesicle that evaginates from a part of the diencephalon (DI). The optic vesicle undergoes invagination to form cup-like structures with two-cell layers: neural retina (NR) in the inner wall and retinal pigment epithelium (RPE) in the outer wall. The optic cup formation of 3D hESC aggregates was initiated by WNT inhibitors that promote rostralization and counteract caudalizing effect of knockout serum replacement in the medium [15]. Short treatment (3 days) of FBS then turns out retinal differentiation of the organoid. Since prolonged FBS treatment enhances NR development but does not support RPE growth, the GSK3β inhibitor is subsequently added in the medium to induce both NR and RPE and morphogenesis of optic vesicle–like structures. The extended culture with N2 supplement–containing medium leads to invagination of the distal portion of the vesicle and the development of the double-layered optic cup structure. In the organoids, rods, cones, interneuron progenitors, and ganglion cells are differentiated and organized into multilayer tissue reminiscent of the postnatal retina. Recently, the retinal organoids have been widely used to understand the molecular mechanisms of photoreceptor-degenerative diseases using patient-derived iPSCs [32]. In addition, engraft of retinal progenitor sheets from the retinal organoids into the subretinal space of the patient is a feasible strategy for cell therapy of eye diseases, since the eye is privileged from immune system and transplantable with relatively safe surgical procedures [33].

### Midbrain organoid

The midbrain constitutes the rostral part of the brainstem controlling the motor ability and is anatomically connected caudally to the cerebellum and rostrally to the basal ganglia and the thalamus/hypothalamus. The mesencephalon (ME) patterning is accomplished by the treatment of WNT, SHH, and FGF8 into NE aggregates [17, 30]. The activation of WNT and SHH signaling promotes the specification of the neural tube into posterior subdivisions, while FGF8 is a key regulator for isthmic organizer. At the early stage, the midbrain organoids contain neuronal progenitor cells expressing a floor plate marker, FOXA2, together with midbrain dopaminergic (mDA) markers, OTX2 and LMX1A. The floor plate progenitors migrate ventrally from the ventricular and intermediate zone into the mantle zone, where mature mDA neurons start to express a dopamine synthetic enzyme and transporter, TH and DAT, respectively. Interestingly, the midbrain organoids under long-term culture show black/brown neuromelanin-like granules, which may protect cells from iron-mediated oxidative stress that is accumulated during aging in the substantia nigra pars compacta of primates, but not in mice [17]. Since PD is typically characterized by degeneration of mDA neurons in the substantia nigra, the midbrain organoid is a primary in vitro model for the PD pathogenesis and drug screening.

### Cerebellar organoid

The cerebellum is essential for motor control including equilibrium and posture and arises from the rhombencephalon (RH). Early FGF2 treatment together with insulin into NE aggregates promotes their caudalization and the formation of isthmic organizer–like structures [18]. Subsequent addition of FGF19 promotes dorsoventrally polarized hindbrain neural tube–like NE structures. The formation of the rhombic lip–like structure is facilitated by sequential addition of SDF1 that is secreted from meningeal cells in embryonic cerebellum. The cerebellar organoids exhibit cerebellar plate neuroepithelium, Purkinje cell, deep cerebellar nuclei, and granule neuron that constitute the cerebellar area. In mice, inhibition of SHH signaling (e.g., cyclopamine) is essential for the cerebellar plate specification, but not necessary in humans [34].

Cerebellar neurodegeneration manifests with symptoms of motor abnormalities including ataxia, difficulty in speaking, and tremor. The cerebellar organoids recapitulate early developmental stage of cerebellar organization. Thus, it is good to model cerebellar diseases in neonatal phase like congenital malformation and neurodevelopmental disorders, such as Dandy-Walker syndrome and Joubert syndrome. Since neurodegeneration in the cerebellum has been observed in Huntington’s disease, the cerebellar organoids are also promising model system for neurodegenerative diseases.

### Spinal cord organoid

Primary sensory information about the external environment is received from the skin and muscle and transmits signals into the spinal cord and up to the brain. Cortical motor signals that are mainly produced from the motor cortex are returned into the peripheral tissues throughout the spinal cord. Thus, the spinal cord is essential for most bodily functions, including speech, sensation, and muscle movement, so damage to the spinal cord devastates the motor abilities and the quality of life of patients permanently. Two-dimensional (2D) differentiation of spinal motor neurons from hPSCs is initiated with dual SMAD inhibition followed by activation of Wnt/β-catenin signaling via GSK3β inhibition (e.g., CHIR-99021) [35]. The combinatorial activation with FGF2, retinoic acid (RA), and SHH accelerates generation of spinal neural progenitor cells that are eventually differentiated into spinal motor neurons with subsequent BDNF and GDNF treatment. Spinal cord organoid protocols have been recently developed by modifying the protocol of the 2D spinal motor neuron induction [19, 36]. To achieve in vitro 3D formation of spinal cord tissue, NE aggregate is induced by single SMAD inhibition and caudalized by GSK3β inhibitor, FGF2, and RA treatment under the suspension culture [19]. Removal of BMP inhibitor and SHH agonist from the original 2D protocol supports generation of wider domains of the spinal cord. Subsequent BMP4 treatment can dorsalize the spinal cord organoid with increasing spinal interneuron in the most dorsal subdomain (dI1 interneuron). Since BMP4 signaling contends with RA-mediated activation of PAX6 that shows lower expression in the dorsal domains, RA removal from the protocol further enhances the dorsalization of the spinal cord organoid. In contrast, ventralization of the spinal cord organoid is promoted by addition of SHH agonist in a dose-dependent manner. Moderate activation (SAG 50nM) accelerates cell differentiation to intermediate domains (p0-p2), whereas the commitment into the most ventral domains (pMN and P3) is enhanced by higher concentration of SHH agonist (SAG 500nM). The p2 intermediate domain is further divided into V2a and V2b subdomains under the control of NOTCH signaling. Subsequent treatment of NOTCH inhibitor (e.g., DAPT) increases and decreases the ratio of V2a and V2b interneurons, respectively. Overall, the spinal cord organoid produced by this protocol displays plasticity of spinal cord domains and can be guided to both dorsal and ventral sides.

Spinal muscular atrophy (SMA) is a genetic neuromuscular disorder that is characterized as degeneration or developmental defect of spinal motor neurons. In particular, neonatal onset of SMA, called Werdnig-Hoffmann disease, is caused by homozygous mutations or deletions in the *SMN1* gene. A recent study demonstrated that the ventral spinal cord organoids from SMA patient–derived iPSCs show decline of the motor neuron differentiation [36]. The depletion of *SMN1* expression activates cell cycle–related genes and promotes re-entry into the cell cycle in the motor neurons. Interestingly, treatment of CDK4/CDK6 inhibition (e.g., PD 0332991) can attenuate the reduction of motor neuron differentiation. Therefore, the spinal cord organoid is a useful tool to investigate the pathological mechanism and development of new medical approaches for neuromuscular disorders.

Myasthenia gravis (MG) is an autoimmune disorder that disrupts transmission of nerve impulse in neuromuscular junctions (NMJs). Despite the potential applications to various neuromuscular diseases, the spinal cord organoid cannot generate skeletal muscle cells that are divergent from mesodermal lineage. Derivation of NMJ organoid was recently accomplished from neuromesodermal progenitors (NMPs) that are bipotent axial stem cells and can be derived from hPSCs with GSK3β inhibitor and FGF2 in 2D culture conditions [37]. NMPs are then switched into low adhesion plates for 3D formation and differentiated into NMJs by neurobasal medium supplemented with mesodermal growth factors: FGF2, hepatocyte growth factor (HGF), and insulin-like growth factor (IGF). At day 5 post 3D induction, NMJ organoid can be matured and maintained in the neurobasal medium without these growth factors. The NMJ organoid displays elongated morphology whereby motor neuronal and skeletal muscle fields are clearly separated. Typical NMJ characteristics including axon projection into skeletal muscle compartment and terminal Schwann cell capping and contractile activity are reproducible in the organoids. Importantly, NMJ organoids treated by autoantibodies from MG patients reduce contractile activity of the muscle and are available to ask how the functional neuromuscular networks are disrupted by etiological agents.

## Complementation with cell types that are rare in brain organoids

Most of the brain and spinal cord organoid protocols are optimized to direct hPSCs into neuroectodermal lineage. Endothelial cells and pericytes develop from mesodermal cells and constitute cerebral vasculature network that carries oxygen and nutrients through the whole brain. Endothelial cells are essential components of the blood-brain barrier (BBB) that tightly regulates the movement of ions, molecules, and cells between blood and brain parenchyma. Microglia cells are brain-resident macrophages and act as the primary immune defense system of the brain. Since the function of BBB and microglia is tightly regulated at human brain pathogenesis, traditional brain organoids that lack these cells are still insufficient.

During embryonic brain development, several cell types, such as interneurons and oligodendrocytes, appear in specific brain areas and migrated into other areas. Dynamics of cell migration is important for establishment of primary excitatory-inhibitory balance and protection of nerve system from brain injury and intercepted in autism spectrum disorder. Unlike surgically isolated brain samples and rodent models, region-specific brain organoids do not contain these migrated cell types. To overcome the issue of depauperate cell types in the organoids, scientists recently have modified the existing protocols to permit the derivation of oligodendrocyte [38], vascular endothelium [39], and microglia-like cell [40] from hPSCs within the brain organoid (Fig. 1c). Thus, we next highlighted recent improvement of the organoid protocols to compensate the lacked cell types.

### Oligodendrocytes

In mouse prenatal brain, oligodendrocyte precursor cells (OPCs) first arise from MGE and anterior entopeduncular area of the ventral TE around embryonic day 12.5 (E12.5) and gradually spread from ventral to dorsal side [41]. Subsequently, OPCs appear from lateral and caudal ganglionic eminences (LGE and CGE) at E15.5. Since the dorsal origin of OPCs starts to appear at postnatal stage, oligodendrocyte-like cells are absent in most of the cortical brain organoids that mimic fetal brain development [10, 20]. In contrast, the ventral forebrain organoids generate a substantial number of OPC-like cells and its assembly with the dorsal organoids may demonstrate the OPC dynamics like the interneuron migration [10, 20].

Although the fusion with the ventral forebrain organoid has potential to establish oligodendrocyte myelination in the cortex, some recent protocols directly induce oligodendrocyte-like cells into the dorsal cortical organoid using growth factors and small molecules governing oligodendrocyte differentiation [38, 42]. Platelet-derived growth factor AA (PDGF-AA), HGF, and IGF1 are essential mitogens to promote proliferation and survival of OPCs. Triiodothyronine (T3) and adenosine 3′,5′-cyclic monophosphate (cAMP) promote oligodendrocyte differentiation of OPCs by inhibiting their proliferation. Biotin catalyzes biosynthesis of fatty acid that is required for myelin formation of oligodendrocyte. Oligodendrocyte-containing cortical organoids were recently developed by adding PDGF-AA, IGF1, and T3 in the cortical spheroid protocol [10, 38]. Food and Drug Administration (FDA)–approved drugs to regulate promyelination (e.g., clemastine and ketoconazole) also support the efficient derivation of oligodendrocyte in the organoid. Another protocol introduces ventral patterning into the cortical spheroids by SHH agonists and enhances oligodendrocyte maturation using PDGF-AA, HGF, IGF1, T3, and cAMP [42]. These oligodendrocyte-containing organoids successfully reproduce substantial expression of oligodendrocyte maturation markers (e.g., MBP) and myelination of surrounding neurons in the organoid.

The Pelizaeus-Merzbacher disease (PMD) is a monogenetic leukodystrophy that is mainly caused by mutations in the PLP1 X-linked gene. The oligodendrocyte-containing organoids from iPSCs of PLP1 point mutation (254T>G) PMD patients exhibit severe reduction of MYRF-positive oligodendrocyte [38]. PLP1 is usually synthesized in the rough endoplasmic reticulum (ER) and transported into the myelin membrane. However, the mutant PLP1 is abnormally accumulated in perinuclear cytoplasm by inhibiting ER-Golgi trafficking and promoting fragmentation of Golgi apparatus and subsequently induces ER stress and apoptosis. Treatment of an inhibitor of protein-kinase-R-like ER kinase attenuates frank perinuclear retention of the mutant PLP1 and increases the oligodendrocyte populations. Overall, the oligodendrocyte-containing organoids contain all three major cell types of brain and recapitulate their cell-to-cell communications that are essential for proper brain development and function.

### Vascular system

The brain organoids can grow up to 4 mm in diameter around 2 months and be maintained around 1 year. Despite their capacity of long-term maintenance, the brain organoids cannot grow bigger than this size due to a limited exchange of oxygen, nutrient and cellular waste in the inner-most regions of the organoid. The absence of a vascular system is fatal to the organoids and leads to the induction of apoptotic cell death with long-term culture. In addition, the stimulation from vascular endothelial cells is essential for the differentiation of neuroprogenitor cells.

One of the first studies to vascularize the brain organoids was to engraft human brain organoids into the mouse brain [43]. The transplantation of the brain organoid onto the cortex of immunodeficient mice exhibited a robust integration of the graft. Interestingly, murine blood vessel started to migrate from host brain into the graft at 1 week of post-implantation, and extensively organized vascular network in the graft at 2 weeks post-implantation. The integrated vascular structure enhanced the progressive maturation of the engrafted organoids and long-term survival. Furthermore, human neurons projected their axons throughout the host mouse brain and establish functional synaptic connectivity with the host neuronal circuit. Therefore, an in vivo engraftment model of the human brain organoid enables us to investigate human brain development and pathogenesis of neuronal diseases under physiological tissue environment.

Functional vascularization of the brain organoids was also modeled in in vitro systems. Under 2D culture, derivation of endothelial cells from hPSCs is initiated by mesodermal formation with WNT activation (e.g., CHIR99021) [44]. Subsequent treatment of BMP4, VEGF, and FGF2 directs the mesodermal cells into endothelial progenitors that are further differentiated into vascular endothelial cells with VEGF-containing medium. The brain organoid and endothelial cells were separately differentiated from the same iPSCs in each culture medium around 1 month and then embedded into porimerized Matrigel droplet with 1:1 mixture of the organoid and endothelial maturation medium [44]. In the Matrigel-coating coculture, CD31-positive endothelial cells formed the tubular structure surrounding the brain organoid. A part of the blood vessel–like tube was integrated toward the brain organoid. However, the vast majority of vascular endothelial cells self-organized severally outside from the brain organoid. Thus, simultaneous generation of endothelial cells with the brain organoids is important for the establishment of functional vascular networks in the in vitro system.

E26 transformation–specific (ETS) family proteins are involved in the transcriptional regulation of genes related to endothelial and hematopoietic differentiation. Among ETS transcription factors, ectopic induction of ETV2 alone sufficiently converts human dermal fibroblasts into vascular endothelial cells [45]. Furthermore, ETV2 overexpression can generate endothelial cells under EB and neuronal differentiation [39], suggesting that ETV2 harbors the capacity for EC formation from various cell types in the absence of essential growth factors for endothelial maturation (e.g., VEGF). Our group previously developed the vascularized brain organoids by mixing ETV2-inducible and non-inducible hESCs [39]. ETV2 activation was initiated by adding low amounts of doxycycline with neuronal induction step (at day2) and fully activated at cortical differentiation stage (at day 18). The ETV2-expressing cells organized the vasculature-like structure that was successfully integrated into the brain organoids. Fluorescein isothiocyanate (FITC)-dextran assay with peristaltic pump demonstrated the existence of a perfusable vascular-like network in the ETV2-induced brain organoid. The vascularization dramatically reduced the apoptotic cell death inside the organoids and supported the increase of the size and long-term maintenance of the organoids. Importantly, the vasculature-like structure displayed a tight junction formation, pericyte production, and astrocytes that are major characteristics of BBB. The malformation of the tight junctions in BBB is an early sign of AD. Amyloid-β (Aβ), an oligopeptide that is deposited in AD patient’s brain, disrupts BBB by digesting extracellular matrix and cell surface components abnormally. The treatment of Aβ_1-42_ oligo disrupted the tight junctions in the vascularized brain organoids, decreasing the perfusability. Furthermore, the transplantation of the vascularized organoid into the mouse brain displayed the functional connection of the human vasculature with the host blood flow network, indicating that in vitro formation of the vascular system is essential not only to mimic physiological environment of the human brain but also for subsequent applications of brain organoids to disease modeling and drug testing.

Human umbilical vein endothelial cells (HUVECs) harbor the property to form capillary-like structures and commonly used primary cells to study the vasculogenesis and angiogenesis. An alternative vascularized organoid can be archived by spontaneously inducing brain organoids with mixture of hESCs and HUVECs [46]. Within the brain organoids, HUVECs show characteristics of brain endothelial cells, such as expression of P-glycoprotein that is absent in HUVEC culture alone. Unlike the ETV2-induced way [39], this method can produce the vascularized organoid without transgenes. However, to generate patient-specific vascularized organoid for disease modeling and drug testing, both HUVECs and iPSCs must be isolated and generated from the same patient.

### Microglia

Organoids cultured in vitro are more vulnerable to the cellular stress and damage than in vivo brain [22]. Microglia plays an important role in repair and remodeling of the CNS by an active immune response and mediates the inflammatory response in a variety of neurodegenerative diseases. Hematopoietic progenitor cells (HPCs) can be induced from hESCs with stage-specific addition of BMP4, VEGF, FGF2, and hematopoietic cytokines [47]. Subsequently, microglia-like cells are differentiated from hESC-derived HPCs in serum-free medium containing CSF-1, IL-34, and TGFβ1. The brain organoids are separately differentiated from hESCs and mixed with the induced microglia-like cells after several months. Interestingly, the coculture experiments demonstrated that the induced microglia-like cells enter the brain organoid and are preferentially accumulated in the injury site with ramified morphology that is essential for transformation to active state of microglia. Unlike the vascular formation, spontaneous induction seems to be not necessary for the establishment of microglia-containing organoids.

Non-guided whole brain organoids are known to produce cell types of mesodermal origin that are represented by expression of myogenin and myosin genes (e.g., MYH3) [5, 7]. Recently, microglia-like cells differentiated from the mesodermal progenitors were found in the non-guided brain organoids by delaying Matrigel embedding and reducing heparin that stimulate neuroectodermal fate commitment [40]. Microglia-like cells from this study displayed the substantial expression of classical markers (e.g., IBA-1) and the morphological change from round shape to ramification. Expression of some markers in the microglia-like cells were not comparable to those in primary adult microglia but were increased with long-term culture of the organoids. In addition, the microglia-like cells isolated from the organoid exhibited the pro- and anti-inflammatory response to lipopolysaccharide (LPS) and dexamethasone, respectively. Since the brain organoid protocols are optimized to recapitulate early embryonic brain development, the generation of the microglia-like cells raises the important question of developmental origin of microglia: whether the microglia can develop in in vivo fetal brain, though the yolk sac is supposedly the major origin of the primitive myeloid progenitors. These questions may be clarified by lineage-tracing approaches to distinguish myeloid cells for microglia or non-microglia. Regardless of the origin, the microglia in the brain organoids will be important tools to study how they regulate the neurodevelopmental process and how they respond to the neurodegenerative damage in brain.

## Systematic comparisons of organoid protocols and fetal brains

Single-cell transcriptional profiling (scRNA-seq) is often coupled with the organoid studies to address the molecular features and heterogeneity of individual cells [7, 8, 10, 14, 20, 38, 39, 42]. scRNA-seq is also a powerful tool to assess the fidelity of cell type specification with their in vivo counterpart regions and in the organoids generated from different protocols [9, 21, 22]. scRNA-seq classified individual cells from the brain organoids into clusters with their molecular features. Each cluster is manually assigned to cell type by single/multiple markers [9, 10, 22, 38, 42, 48] or gene signatures from Gene Ontology and reference transcriptome profiles [7, 8, 14, 20, 39]. Although the cluster labeling differs among different research groups, the single-cell analyses similarly identified the typical CNS cell types, including neurons and astrocytes in the brain organoids. Interestingly, single-cell transcriptome data further divides the cell types into several subtypes that display distinct gene expression patterns. SOX2, VIM, and HES1 are typically present in neural stem cells, including the dividing neuroprogenitors, and radial glia cells. In addition to these well-defined cell types, our group identified several uncharacterized glia cell types that express genes related to proteoglycan, cilia assembly, and BMP signaling [21]. These cell types are also detected from human fetal brain. Although their function in brain development is still unclear, the scRNA-seq analysis can clarify the presence of unique cell types in the organoid and brain.

Current brain organoid protocols utilize different combinations of signaling inhibitors and were previously categorized by their cortical patterning level: non-directed [5], least directed [4], directed [10], and most directed [20]. Despite the stringency of the cortical direction, all protocols exhibit broad expression of FOXG1 forebrain markers and similar cell composition [21, 22]. However, compared to primary brain sample, cells from the organoid highly express genes related to glycolysis and endoplasmic reticulum (ER). Although primary brain shows laminar structure of the neurogenesis, organoids dissolve the cortical layers and intermix both progenitors and neurons with prolonged culture. These differences between primary brain and organoid may be caused by metabolic stress from organoid environment (e.g., lower oxygen) that activates glycolysis and ER-related genes and impairs the cell-type specification. The deterioration of neuronal development can be rescued by adapting organoids to in vivo environment, such as transplantation. The integration of vascular network may reduce the cellular stress and leads to proper cell type specification [43]. In vitro derivation of vasculature in the organoid is also helpful for the maturation of neuronal cells [39]. Therefore, the attenuation of the cellular stress is essential for the application of the brain organoid to studies of brain developmental processes, cell type–specific diseases, and cell-to-cell interactions.

## Improvement of long-term culture and organoid survival

In addition to the induction of vasculogenesis, researchers have made an effort to ameliorate interior hypoxia and nutrient starvation of the organoids by retaining scalability of in vitro system. One of the advanced approaches is slicing of the brain organoid into a disk shape that allows the exposure of the innermost regions to the external medium environment [49]. After the organoids grow to 1.5-mm diameter, 5000-thick slices are isolated from the middle plane of the organoid by a vibratome. The disk-shaped organoids can obtain oxygen and nutrients from both top and bottom surface and expand to horizontal and thickness axis. The organoids are routinely every 4 weeks and display cytoarchitectures of late developing brains, such as upper and deep cortical layers. Another approach adapts the organotypic slice to air-liquid interface that also improves neuronal survival and morphology [48]. Importantly, the sliced organoids are easily applicable to subsequent experiments including imaging analysis and multiple electrode arrays. Therefore, the slicing methods are potentially useful to examine maturation process of embryonic brain and late-onset neurodevelopmental diseases. In contrast, it is still unclear how slicing procedure affects brain developmental program. Although most of the studies have been implemented by microglia-lacking organoid, there remain questions regarding the induction of neuroinflammation and brain injury responses by the slicing.

## Ethical concerns of brain organoids for biomedical research

Along with the emergence and improvement of 3D brain culture systems, it has been concerned that lab-grown brain organoids become conscious like premature babies [50]. The action potential activity is detectable within 1 month, but discrete and infrequent. However, the firing rate and the burst frequency are increased over the period of several months. Spontaneous synchronization of spikes appears in 2-month organoids and starts to be oscillated after 4 months. Importantly, long-term cultured human brain organoids (> ~6 months) exhibit the firing pattern that resembles human preterm neonatal electroencephalogram (EEG). These observations give rise to ethical questions or concerns whether the application of conscious brain organoids to biomedical research is legitimate or not. Although EEG-like coordinated activity wave is one of the features in the conscious brain, there is no common way to determine the consciousness in the organoid. Whereas the consciousness can be evaluated by response to pain in patients, it is unclear how the organoids feel pain. The consciousness emerges in human brain at around 24 post-conception weeks (pcw). However, previous comparative transcriptome analyses roughly estimated that 6-month-old organoids equal to fetal brains at 16–21 pcw [21]. Even though guideline about the usage and welfare of lab-grown brain tissues should be discussed at this stage, consciousness in the brain organoid is still a debatable topic and may require additional evidences for further discussions.

## Perspective and summary

In conjunction with the brain organoids, the generation of other peripheral tissues has been archived and refined. Most of the organ functions are governed by the nervous system with inputs from the brain and spinal cord to peripheral neurons. Amyotrophic lateral sclerosis (ALS) patients suffer from the abnormality of heart electrical pacing, detoxification, and breathing by the functional defect of motor neurons in the central nervous system (CNS). Neuropathy exhibits the symptom of chronic liver disease and kidney failure with the damaged peripheral nervous system. Stomach and intestinal functions are susceptible to anxiety and stress in the brain. Unlike in vivo organs, the peripheral tissue organoids do not have neuronal cells and are not available to understand how the tissue functions are controlled by the nervous system and invaded by multiorgan diseases. The fusion culture system of brain organoid allows axonal and neuronal projection into the other types of organoids [14]. The coculture with sections of spinal column and paraspinal muscles demonstrates that the spinal cord is innervated by axon tract from the organoid, and extracellular stimulation to the organoid evokes muscle contractions [48]. Multi-organ-on-a-chip technology is another scalable tool to connect multiple organoids with microfluidics and recapitulate physiological interactions. The incorporation of biological and physical sensors with the multi-organ-on-a-chip promotes continual measurements of tissue behaviors and secreted soluble biomarkers [51]. Thus, the establishment of biomemetic scaffold connecting multiple types of organoids will enable dissecting the complexity and crosstalk of the multiorgan diseases.

Together, the organoid technologies have rapidly progressed and opened new avenues for biomedical applications, but are still growing. In particular, further expansion and maturation of the brain organoids to late fetal and perinatal stage brain development, at which synaptic pruning, myelination, and cortical layer formation begin, are required. The multidisciplinary approaches to build biomimetric organoid culture system are also exciting opportunities. The “vascularization,” “immunization,” and “neuronization” will improve the maturation of organoid systems and be more amenable to in vitro pathological researches and eventual regenerative medicines.
